# Prodigiosin Production by *Serratia marcescens* UCP 1549 Using Renewable-Resources as a Low Cost Substrate

**DOI:** 10.3390/molecules15106931

**Published:** 2010-10-08

**Authors:** Helvia W. Casullo de Araújo, K. Fukushima, Galba M.Campos Takaki

**Affiliations:** 1 Doutorado da Rede Nordeste de Biotecnologia (RENORBIO)-Universidade Católica de Pernambuco (UNICAP) Recife, PE, Brazil; E-Mail: hwcasullo@ig.com.br (H.W.C.A.); 2 Núcleo de Pesquisas em Ciências Ambientais (NPCIAMB), Universidade Católica de Pernambuco (UNICAP) 50.050-900 Recife, PE, Brazil; 3 Departamento de Química, Universidade Estadual da Paraíba (UEPB), Campina Grande - PB, Brazil

**Keywords:** prodigiosin, *Serratia marcescens*, corn steep liquor, cassava liquid waste, mannitol

## Abstract

A new strain of *Serratia marcescens* UCP1459 isolated from a semi-arid soil produced the natural red pigment prodigiosin, characterized by an uncommon pyrrolylpyrromethane skeleton. Prodigiosin is a promising drug due to its reported antifungal, immunosuppressive and anti-proliferative activities. The objective of this work was to indentify a suitable medium to simultaneously enhance *S. marcescens* growth and pigment production using renewable resources obtained from industrial wastes. *S. marcescens* produced the highest level of prodigiosin (49.5 g/L) at 48 h of cultivation using 6% “*manipueira*” (cassava wastewater) supplemented with mannitol (2%) at pH 7 and 28 °C. Carbohydrates in “*manipueira*” and mannitol play a role in the enhanced cell growth and prodigiosin production. The purified pigment extracted from the biomass was analyzed by mass spectrophotometry and showed the expected molecular weight of 324 Da corresponding to prodigiosin. In conclusion, we have successfully designed a new, economically feasible medium supporting enhanced S. marcescens growth and a high yield production of prodigiosin.

## Introduction

**Figure 1:**
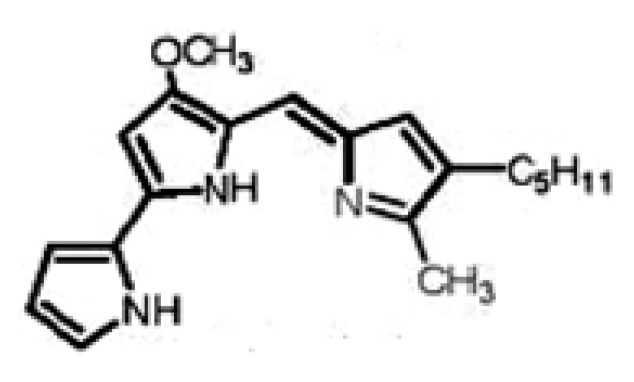
Chemical Structure of Prodigiosin [3,4].

Prodigiosin (5[(3-methoxy-5-pyrrol-2-ylidene-pyrrol-2-ylidene)-methyl]-2-methyl-3-pentyl-1*H*- pyrrole) is a secondary metabolite alkaloid with a unique tripyrrole chemical structure. It is a red pigment isolated from a few species such as *Serratia*, *Pseudomonas *and *Streptomyces* [1,2]. It has three rings forming a pyrrolylpyrromethane skeleton with a C-4 methoxy group, a molecular formula C_20_H_25_N_3_O and a molecular weight of 323.44 Da (Figure 1) [2,3,4]. It is sensitive to light and insoluble in water. It is moderately soluble in alcohol and ether, and soluble in chloroform, methanol, acetonitrile and DMSO [5,6]. 

*Serratia marcescens* is a rod-shaped, Gram negative, facultative bacterium belonging to the Enterobacteriaceae family and characterized by its ability to produce the red pigment prodigiosin [5]. Chromogenic species are usually isolated from the environment from water, soil, plants or insects. Ten species of *Serratia *have been described, of which only three are capable of producing prodigiosin: *S.* *plymuthica, S. rubidaea* and some biogroups of *S. marcescens* [6,7]. *S. marcescens* is a facultative microorganism and therefore the pigment is produced under both aerobic and anaerobic conditions [7]. 

However, pigmentation is only present in a small percentage of isolated cultures among different S*.* *marcescens* strain. Pigment production is highly variable among species and depends on many factors such as species type incubation time, pH, carbon and nitrogen sources and inorganic salts [8,9]. Some of the strains, not just the chromogenic ones, constitute a real threat in hospitals. 

Although prodigiosin has no known defined role in the physiology of the strains in which it is produced, it has antifungal, antibacterial and antiprotozoal activities, and thus may have potential clinical utility [10]. Both prodigiosin and its synthetic derivatives have potent and specific immunosuppressive activity, with new targets that are clearly distinct from those of other drugs [11,12].

Many types of differential and selective media have been used for *Serratia* growth and prodigiosin production. Regular prodigiosin production has been carried out in nutrient broth containing sesame seeds, maltose broth, peptone glycerol broth [1], and a medium patented by Nakamura and Kitamura [13] containing 2% sodium oleate.

However, it would be desirable to design a new nutritious and economically cheap medium to enhance S. marcescens growth and prodigiosin biosynthesis. In this context, we have observed the importance of agroindustrial cassava wastewater and corn steep liquor as renewable substrates. The wastewater from industrial cassava processing has a huge adverse impact on the environment [14] because it contains a large amount of cyanogenic glucosides (linamarin and lotaustralin), which can be hydrolyzed to hydrocyanic acid (HCN) by an endogenous enzyme (linamarase), when the plant tissue is damaged by harvesting and processing during its preparation as a food product [15,16]. In this study, the substrate used was cassava (*Manihot esculenta *Crantz) liquid waste (“*manipueira*”). Cassava is of great economic importance to several towns in the northeast of Brazil, where its consumption (in terms of carbohydrate content) exceeds that of other crops. In the present study we investigated the influence of agroindustrial cassava liquid waste and corn steep liquor with or without mannitol supplementation as a low-cost medium for *S.* *marcescens* growth and effective production of prodigiosin.

## Results and Discussion

### Effect of agroindustrial substrates on growth and pigment production

**Figure 2:**
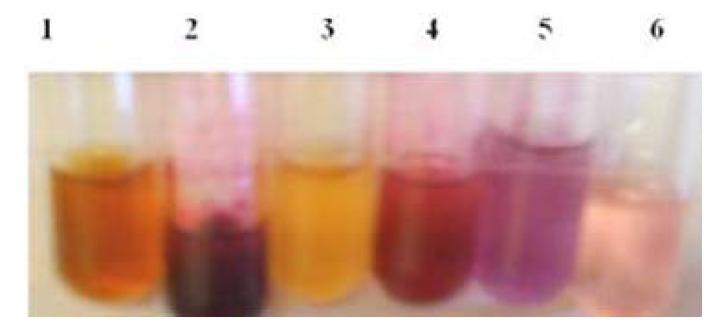
*S. marcescens *growth and prodigiosin production in different media after 48 h at 28 °C. 1 (CSMM), 2 (MM), 3 (CSM), 4 (CWMM), 5 (CWM), 6 (LBGM).

Prodigiosin production by *S.* *marcescens* UCP 1549 was compared for six culture media (Table 1 and Figure 2). 

**Table 1:** Colony color, biomass and prodigiosin production by *S. marcescens *in different media for 48 h at 28 °C.

Medium	Colony color	Biomass (g/L^-1^)	Prodigiosin (mg/L)
Corn steep mannitol medium (CSMM)	Orange	5.679	37500
Mannitol medium (MM)	Dark red	5.463	34000
Corn steep medium (CSM)	Yellow	3.465	25600
Cassava waste mannitol medium (CWMM)	Red	7.562	49500
Cassava waste medium (CWM)	Light red	3.654	27000
Luria Bertani glucose medium (LBGM)	Rose	2.865	13500

The maximum biomass yield was obtained in CWMM, followed by CSMM, MM, CWM, CSM and LBGM. Biomass pigmentation varied from dark red to rose, indicating prodigiosin production at 28 °C. However, the colony color changed to yellow with corn steep liquor as nitrogen source in the medium for both CSM and CSMM. Glucose-supplemented LB medium led to a large decrease in prodigiosin production at 28 °C.

Cassava is widely used in human and animal nutrition and as a raw material for several industrial products, the most important of which are cassava flour, cassava starch and sour cassava starch. The liquid waste from industrial cassava processing contains a large number of pollutants and has a significant adverse environmental impact [14]. Natural “*manipueira*” could be an appropriate substrate and is a promising source of nutrients for biosurfactant production by selected isolates [17]. Our growth and pigment production results are corroborated by Andrade *et al*., who considerers “*manipueira*” as a promising nutrient source when supplemented with mannitol [18]. 

Corn steep liquor is composed of 40% nitrogen and contains amino acids, vitamins, K, P, Ca, S, Na, Fe, Zn, Mn, Cu, Cr, Mo, Se and Co, and lower level of carbohydrates, and consequently offers a rich source for culture media. In fact, *S. marcescens *produced a higher amount of biomass in this medium, but the results clearly showed that this did not significantly enhance pigment production and in fact, the addition of corn steep liquor caused a reduction in prodigiosin production which could be due to catabolite repression.

Cang *et al*. [19] used mineral medium supplemented with ethanol as a carbon source and obtained good results for prodigiosin production after 72 h at 28 °C. However, no significant prodigiosin production was observed for other carbon sources such as rhamnose and lactose, although glucose addition increased prodigiosin to afford 5 mg/L at 48 h [20]. 

The maximum pigment yield was obtained with CWMM, followed by CSMM. The utility of cassava liquid waste as substrate was much higher than that of corn steep liquor. Cassava liquid waste was more efficient in inducing pigment production compared to MM and LBGM. The biomass yields for CSMM and MM were largely similar, but prodigiosin production was lower in the latter (Table 1). Similar results were observed in CSM and CWM with regard to the amount of product produced. However, we detected light yellow extract in CSM, which indicated the pigment synthesis is blocked (Figure 2).

On account of its efficiency, *S. marcescens* has higher potential prodigiosin production for application in the manufacture of medicinally important products. In the present study, six different culture media were investigated for *S. marcescens* growth and prodigiosin production potential to identify possible low cost substrates. Cassava wastewater [17] and corn steep substrates [21] have rich and effective compositions that contribute to prodigiosin production.

From the results obtained, it is possible to deduce that the most probable reason for enhanced bacterial growth and/or lower pigment production is related to the carbon sources and the final pH of the medium. Literature results for prodigiosin production are shown in Table 2. Harned obtained a yield of 5 mg/L using a medium with glucose as a carbon source [20]. Cang *et al*. [19] obtained a yield of 3,000 mg/L for a medium containing ethanol as carbon source [19]. Giri *et al. *[1] obtained maximum yields of 16,680 and 38,750 mg/L for sesame and peanut seed broth as culture media respectively. Song *et al.* [2] used casein as the carbon source for prodigiosin production and obtained 4,280 mg/L. More recently, Venil and Lakshmanaperumalsamy [22] used a factorial design for optimization of prodigiosin production by *S. marcescens *and obtained a maximum yield of 1,397 mg/L. Nitschke *et al*. [17] reported that cassava liquid waste gave the best biosurfactant yield among various carbon sources tested. The results obtained revealed enhanced prodigiosin production in all the media studied. In our case, cassava liquid waste supplemented with mannitol gave a better yield compared to other media used.

**Table 2:** Comparison of prodigiosin production by *S. marcescens* in different culture media.

Culture medium	Prodigiosin ( mg/L )	Reference
Glucose	5	[20]
Ethanol	3,000	[19]
Sesame seed broth	16,680	[1]
Peanut seed broth	38,750	[1]
Casein	4,280	[2]
(NH_4_)_2_PO_4_^+ ^salt	1,397	[22]

### Kinetics of S. marcescens growth and prodigiosin production

**Figure 3:**
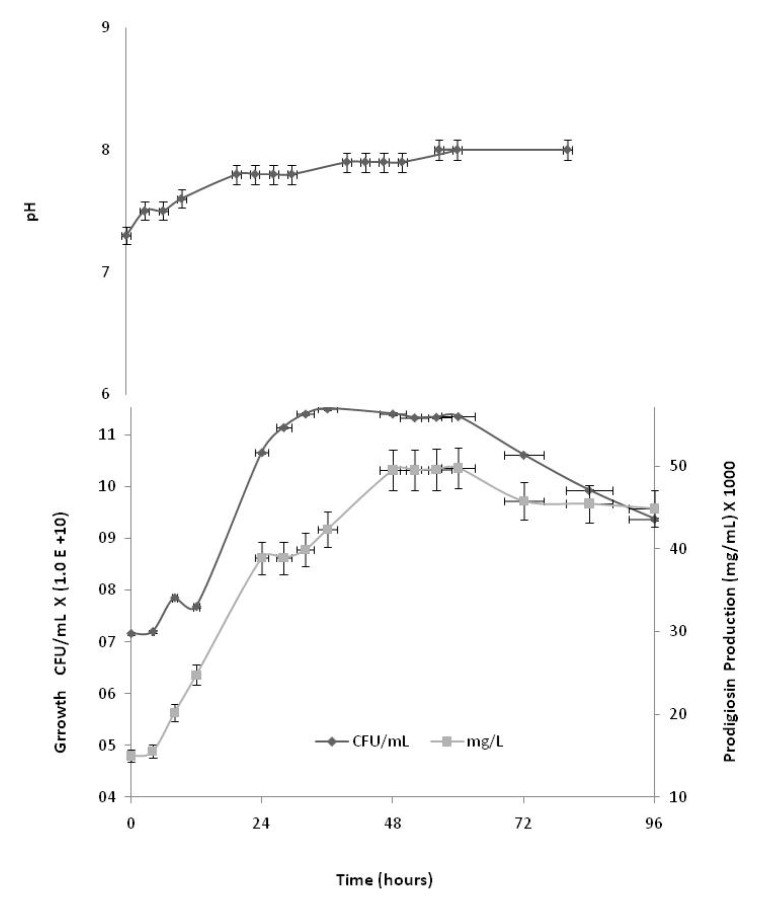
Growth kinetics, pH and prodigiosin production by *S. marcescens* in CWMM at 28 °C for 96 h.

Figure 3 shows *S.* *marcescens *growth, pH and prodigiosin production (mg/L) in CWMM. A diauxic lag phase was observed to occur at 12 h. After which exponential growth was observed up to 36 h. The specific growth rate was μ_esp_ = 0.36 h^-^^1^, the generation time was *T_G_* = 1.88 h, 43,000 mg/L prodigiosin was produced, and the maximum yield was observed at 48 h. In the stationary phase prodigiosin production decreased with increasing pH. An appropriate fermentation time for prodigiosin production is thus approximately 48–72 h. Prodigiosin is also produced at lower temperatures such as 25 and 30 °C. It is possible temperature affects the activity of one or more enzymes involved prodigiosin synthesis. At temperatures over ≥ 30 °C, a particular enzyme in the prodigiosin synthesis pathway may lose activity. A number of other factors can affect the temperature regulation of synthetic reactions. 

Temperature can affect how well at cell absorbs the material needed for pigment synthesis or how quickly it is degraded. In this case the strain obtained from an arid soil produced pigmentation at 28 °C, in agreement with results reported by Chang *et al*. [23] prodigiosin production by *S. marcescens* strain 389 in a mineral medium. 

Mannitol in the cultivation medium was a decisive factor in maintaining the pH, which reached stable values of 7.0 at the beginning of the stationary growth phase, probably owing to the production of secondary metabolites such as prodigiosin. In the presence of carbohydrates from cassava liquid waste, there was an increase in pigmentation in the initial hours of cultivation, reached 49,500 mg/L at 48 h (Figure 3). A strong characteristic dark red color indicated that prodigiosin was being formed by *S.* *marcescens *owing to the CWMM carbon source.

### Prodigisiosin extraction and identification

**Figure 4:**
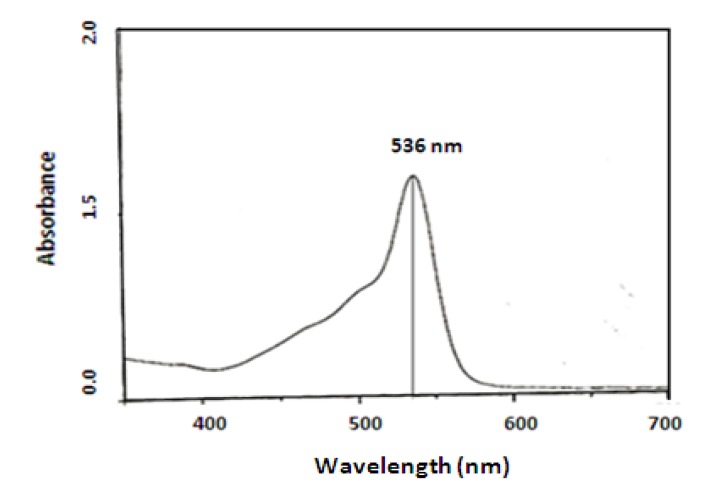
Absorbance spectrum of prodigiosin purified by *Serratia marcescens* UCP 1549.

**Figure 5:**
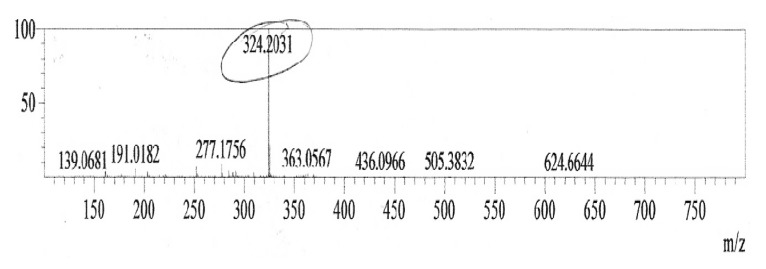
Mass spectrum of the purified red pigment produced by *S. marcescens* UCP 1549.

According to Melo *et al.* [24] pigment extraction using acidic and basic solutions does not eliminate impurities, even after purification by chromatography. We used the methodology described by Nakashima *et al*. [25] to purify the pigment produced by *S. marcescens *UCP 1549. The crude prodigiosin extract obtained (CWMM) was purified by TLC. The extract had the same *R_f_* value (0.9) as a prodigiosin reference material. The pigment was analyzed by UV spectrophotometry and mass spectrometry. The maximum UV absorbance was observed at 536 nm (Figure 4), corresponding to prodigiosin [25], in agreement with results for prodigiosin purified from *Serratia* sp KH – 95 [2]. Mass spectrometry revealed a molecular weight of 324 Da for the purified pigment (Figure 5), in agreement with results reported by Song *et al. *[2] for prodigiosin.

## Experimental

### Microorganism and culture conditions

A pigmented strain of *Serratia marcescens* UCP 1549 was isolated from a semi-arid soil around banana trees belonging to the culture collection of the Nucleus of Research in Environmental Sciences (NPCIAMB/PE), Catholic University of Pernambuco, Brazil. The strain was registered with the World Federation for Culture Collections and was maintained for 1 month at 5 ^o^C on nutrient agar medium. 

To obtain an appropriate amount of bacterial biomass, the strain was transferred to a 250 mL Erlenmeyer flask containing Luria Bertani broth medium (LB, 100 mL) and was incubated at 28 °C for 24 h at 150 rpm and pH 6.5 as a prodigiosin control. Pigment production was carried out using solid medium (20 mL) in Petri dishes (100 × 95 mm) containing *S. marcescens *(2 mL)*. *Experiments were conducted used the following media: 6% cassava wastewater supplemented with 2% mannitol (CWMM); 6% corn steep liquor supplemented with 2% mannitol (CSMM); 6% cassava wastewater (CWM); 6% corn steep liquor (CSLM); and 2% mannitol (MM); all containing 1.5% agar. The plates were incubated at 28 °C for 48 h until the appearance of red pigmentation. 

### Pigment extraction and isolation

Pigmented cultures were scraped from the surface of the Petri dishes. The biomass was extracted in chloroform/methanol mixtures of increasing polarity (2:1, 1:1 and 1:2 v/v) and then pure methanol over a period of 24 h [25]. The crude extract was evaporated to dryness and the amount of pigment obtained on a dry weight basis was calculated.

### Kinetics of growth and prodigiosin production

The specific growth rate (μ_esp_) and generation time (*T_G_*) were determined according to the formula described by Pirt [26]:

(µ_esp _) = (ln*X* – ln*X*_0_) / (*T* – *T*_0_) (1)

where *X* is growth in the exponential phase, *X_0_* is growth in the early exponential phase, *T* is time to the exponential growth phase, *T_0_* is the initial time at which the exponential phase occurs and:

T*_G_* = ln2/μ_esp ._ (2)

The prodigiosin production was measured by spectrophotometry at 536 nm using ethanol extract from biomass of *S. marcescens *and was expressed in mg/L.

### Purification and chemical structure of the pigment

The crude extracts were pre-purified TLC in Si 250F silica gel plates using 9:1 chloroform/methanol as the solvent system. The pigment of interest was removed and purified via column chromatography (50.0 × 1.0 cm) using silica gel as adsorbent. The pigment was dissolved in chloroform (100 mL), which was then removed by rotary evaporation. The resulting product was identified as prodigiosin by UV-visible spectrophotometry in the range 700–200 nm in 95% ethanol and subsequent mass spectrometry using methanol.

## Conclusions

Cassava wastewater supplemented with mannitol was at good medium for prodigiosin production by *S. marcescens*. The carbon/nitrogen ratio is a crucial parameter for prodigiosin accumulation. The high amount of carbohydrates and nitrogen in cassava liquid waste may contribute to prodigiosin accumulation and significant growth on raw effluent was observed. However, mannitol addition can improve this raw substrate for *S. marcescens* growth. This suggestion is based on the levels of carbohydrate, nitrogen (ammonium and urea), phosphorus, lipids, iron, potassium, sulfur, boron, copper, calcium, and manganese in cassava wastewater [17], as a substrate for higher yield prodigiosin production. *S. marcescens *UCP 1549 demonstrated an ability to grow on HCN in cassava liquid waste. The results for this new *S. marcescens* strain are quite promising in terms of increasing the scale of prodigiosin production using agroindustrial effluent and contributing to the removal of cassava wastewater pollutants. In addition, “*manipueira*” use could reduce the costs associated with this pollutant and thus could contribute to environmental improvement. 
